# Aspects épidémiocliniques et suites judiciaires des abus sexuels chez les mineurs à Monastir, Tunisie

**DOI:** 10.11604/pamj.2021.38.105.21766

**Published:** 2021-02-01

**Authors:** Rim Ben Soussia, Rim Gniwa Omezzine, Walid Bouali, Mouna Zemzem, Sarra Bouslah, Lazhar Zarrouk, Lotfi Gaha

**Affiliations:** 1 Service de Psychiatrie, Hôpital Universitaire Tahar Sfar, Mahdia, Tunisie,; 2 Service de Psychiatrie, Hôpital Universitaire Fattouma Bourguiba, Monastir, Tunisie

**Keywords:** Abus sexuels, mineurs, poursuite judiciaire, Sexual abuse, children, legal action

## Abstract

**Introduction:**

les abus sexuels chez les mineurs constituent une réalité troublante et un problème majeur de santé publique. En effet, il s´agit d´un sujet qui a été longtemps considéré comme tabou, aux conséquences dramatiques sur la santé physique, psychique et le bien-être social des victimes. L´objectif est de préciser les aspects épidémiologiques et cliniques des abus sexuels chez les mineurs, et d´en étudier les aspects juridiques.

**Méthodes:**

une étude rétrospective descriptive a été réalisée à la consultation externe de pédopsychiatrie à l´hôpital universitaire de Monastir durant la période de 12 ans et 6 mois. Nous avons inclus tous les enfants dont l´âge était inférieur à 18 ans et chez qui une agression sexuelle a été suspectée ou retenue.

**Résultats:**

au total, 93 mineurs victimes d´abus sexuels ont été colligés. L´âge moyen des mineurs était de 10 ans avec un écart-type de 3,9 ans. Le sex-ratio (G/F) était de 0,9. Pour les modalités de contact sexuel, les attouchements sexuels étaient les plus fréquents dans 47,3% des cas. La majorité des abuseurs étaient de sexe masculin (93,5%). Dans plus que la moitié des cas (53,8%), ils étaient une personne de l´entourage de l´enfant et dans 28% cas, l´abus était intrafamilial. L´évaluation psychiatrique initiale a objectivé des troubles mentaux dans 70% des cas.

**Conclusion:**

l´abus sexuel est un champ très vaste des agressions de nature et d´intensité très diverses. Même si l´impact physique de cet acte peut manquer dans certaines formes d´abus sexuel, l´impact émotionnel et psychologique est omniprésent avec des expressions cliniques variables.

## Abstract (en)

**Introduction:**

Child sexual abuse is a disturbing reality and a major public health problem. Indeed, it is a subject that has long been treated as taboo, with dramatic consequences on physical and mental health as well as on social wellbeing of the victims. The purpose of this study was to highlight the epidemiological and clinical features as well as the legal aspects of child sexual abuse.

**Methods:**

we conducted a retrospective descriptive study at the psychiatric outpatient service of the University Hospital of Monastir over a period of 12 years and 6 months. All children younger than 18 years diagnosed with suspected or confirmed sexual assault were included.

**Results:**

a total of 93 children, victims of sexual abuse were enrolled. The average age of patients was 10 years, with a standard deviation of 3.9 years. Sex-ratio M/F was 0.9. The most common sexual contact was touching (47,3% of cases). The majority of abusers were male (93,5%). In more than half of the cases (53,8%) they were relatives of the child and in 28% of cases intra-familial abuse was reported. Initial psychiatric assessment showed mental disorders in 70% of cases.

**Conclusion:**

sexual abuse is a very broad field of violence which varies widely in nature and intensity. Although, in some types of sexual abuse occasionally there is a lack of physical traces, the emotional and psychological impact is ubiquitous and characterized by numerous clinical manifestations.

## Introduction

Les abus sexuels chez les mineurs constituent une réalité troublante et un problème majeur de santé publique. En effet, il s´agit d´un sujet qui a été longtemps considéré comme tabou, aux conséquences dramatiques sur la santé physique, psychique et le bien-être social des victimes [1]. L´OMS, cité par plan international, estimait en 2002 que 150 millions de filles et 73 millions de garçons avaient été victimes d´abus sexuels dans le monde [2]. En Tunisie, et avec la liberté de la presse reconquise depuis janvier 2011, on a noté ces dernières années, une augmentation considérable des signalements au judiciaire avec pour corollaire une demande accrue d´examens médico-légaux, en particulier des expertises psychiatriques [3]. Nous adoptons dans le cadre de ce travail la définition de l´OMS: « l´exploitation sexuelle d´un enfant implique que celui-ci est victime d´un adulte ou d´une personne sensiblement plus âgée que lui, aux fins de la satisfaction sexuelle de celui-ci. Le délit peut prendre plusieurs formes: appels téléphoniques obscènes, outrage à la pudeur, voyeurisme, viol, inceste, prostitution des mineurs, etc. » Dans ce cadre, les objectifs de notre étude étaient de préciser les aspects épidémiologiques et cliniques des abus sexuels chez les mineurs, et d´en étudier les aspects juridiques.

## Méthodes

**Type d´étude**: il s´agit d´une étude rétrospective descriptive concernant réalisée à la consultation externe de pédopsychiatrie à l´hôpital universitaire de Monastir durant la période allant du 1er Juillet 2004 au 31 Janvier 2016. Nous avons inclus tous les enfants dont l´âge était inférieur à 18 ans et chez qui une agression sexuelle a été suspectée ou retenue.

**Paramètres étudiés et analyse des données**: le recueil des données s´est fait à partir du dossier clinique et judiciaire selon deux fiches à savoir 1) une fiche des données épidémiocliniques décrivant l´âge, le sexe, le profil familial et socioculturel de l´enfant, les circonstances de l´agression et les caractéristiques de l´agresseur, le type de contact sexuel, les lésions éventuelles retrouvées au cours de l´examen physique. 2) une fiche des données judiciaires: ils ont été recueillis auprès de la délégation régionale de la protection de l´enfance de Monastir, et du bureau du juge de famille au tribunal de première instance de Monastir. Les diagnostics cliniques ont été retenus selon les critères du DSM-5 (Manuel Diagnostique et Statistique des troubles mentaux de l´Association Américaine de Psychiatrie, 5^e^ version). L´analyse statistique s´est faite avec le logiciel SPSS dans sa 2^e^ version.

## Résultats

**Le profil sociodémographique de la population étudiée**: durant la période d´étude, 93 mineurs victimes d´abus sexuels ont été colligés. L´âge moyen des mineurs était de 10 ans avec un écart-type de 3,9 ans. La population âgée de 12 à 18 ans était la plus touchée avec un taux de 60,2%. Le sex-ratio (G/F) était de 0,9. La totalité de l´échantillon vivaient en milieu urbain. Les parents des enfants étaient divorcés dans 16% des cas. Chez 31,6% des enfants, il y avait une histoire de maltraitance psychologique ou physique intrafamiliale antérieure à l´agression sexuelle.

**Figure 1:**
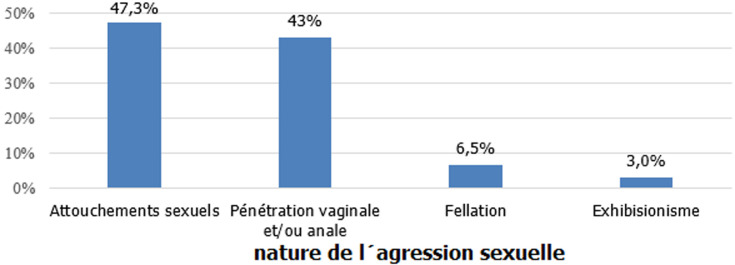
répartition selon la nature de l'agression sexuelle

**L´abus sexuel: nature et contexte**: les différents types de sévices sont illustrés dans la Figure 1. Il s´agissait essentiellement des attouchements sexuels dans 47%. La majorité des abuseurs étaient de sexe masculin (93,5%). Dans plus que la moitié des cas (53,8%), ils étaient une personne de l´entourage de l´enfant et dans 28% cas, l´abus était intrafamilial. Au cours de l´abus sexuel, 26% des mineurs ont été victimes de violence physique ou morale (harcèlement morale, menace...).

**Symptomatologie clinique**: l´agression sexuelle était le motif de consultation le plus fréquent en pédopsychiatrie chez 47% des enfants, dont 44% consultaient dans le cadre d´une expertise médicolégale. Quant à l´évaluation psychiatrique initiale, elle a objectivé des troubles mentaux dans 70% des cas. Il s´agissait essentiellement d´un trouble dépressif caractérisé et d´un trouble de l´adaptation avec des taux respectifs de 32% et 21% (Tableau 1).

**Tableau 1:** troubles psychiatriques retenus initialement chez les victimes

Trouble mental	Nombre (N)	Pourcentage(%)
Trouble dépressif	30	32,5
Trouble de l´adaptation	19	20,4
Trouble de stress post-traumatique	11	11,8
Trouble de conduites	05	5,3
Pas de trouble objectivé	28	30
**Total**	**93**	**100**

**Figure 2:**
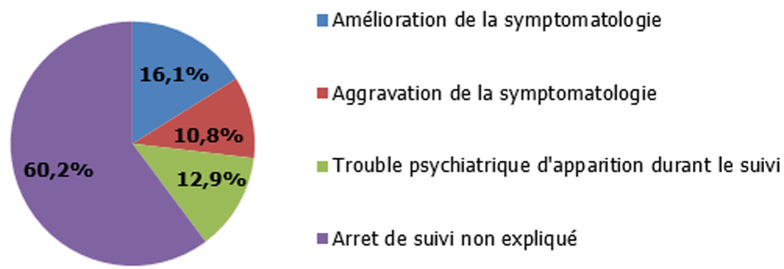
profil évolutif à court et à moyen terme des victimes

**Données concernant la prise en charge**: une prise en charge spécialisée a été fournie pour chaque enfant diagnostiqué avec un trouble mental. En dehors des situations d´expertise médicolégale ou la procédure sociojuridique a été déjà mise en marche, un signalement a été fait auprès de la délégation régionale de la protection de l´enfance du gouvernorat de Monastir pour tous les enfants suspectés avoir été victimes d´agression sexuelle, soit 56% de notre population d´étude. Par ailleurs, 60% de ces enfants ne sont plus présentés en consultation au bout d´un mois de suivi psychiatrique. Ce suivi n´a pu être maintenu à moyen terme que chez 40% des patients. Chez ces derniers, le profil évolutif à court et à moyen terme (au bout de deux mois à un an du suivi) n´a pas montré une prédominance d´un mode évolutif parmi les trois alternatives constatées chez ces patients ; aggravation ou amélioration de la symptomatologie initiale et l´apparition secondaire d´un trouble mental (Figure 2). Concernant l´évolution à moyen terme a été émaillée par l´apparition d´un trouble psychiatrique chez 12 enfants. Les troubles de conduites graves et le trouble bipolaire étaient les diagnostics les plus notés avec des taux respectifs de 7,5% et 4,3% (Tableau 2).

**Tableau 2:** les troubles mentaux retenus secondairement au cours du suivi

Trouble mental	Nombre (N)	Pourcentage (%)
Trouble de conduites graves	07	7,5
Trouble bipolaire	04	4,3
Trouble psychotique	01	1,1
**Total**	**12**	**12,9**

**Figure 3:**
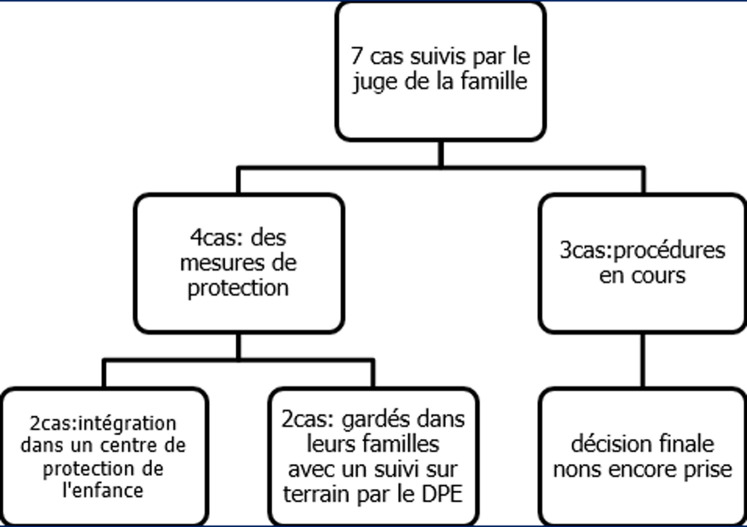
schéma récapitulatif des mesures de protection prises par le juge de la famille

**Suites judiciaires**: au total, 25 enfants (26.8%) seulement ont été suivis par le délégué de protection de l´enfance et 7 enfants (7,5%) parmi eux ont poursuivi les procédures judiciaires avec le juge de la famille. Les suites judiciaires des affaires relatives à ces agressions ont été obtenues auprès du bureau du Juge de la famille au tribunal de première instance de Monastir. D´après cette source, parmi les sept victimes suivies (Figure 3): 1) trois affaires sont toujours en cours malgré l´écoulement d´une période de deux ans ou plus depuis la survenue de l´agression et l´évaluation médicolégale. Ceci serait en rapport avec la surcharge en dossiers à traiter et les délais prolongés des enquêtes sociales et des procédures administratives au tribunal. 2) dans les quatre cas restants les mesures de protection ont été prononcées par le juge. Il s´agissait pour deux d´entre eux d´intégrer la victime dans un centre de protection dans un but d´éloignement de l´enfant de son milieu familial jugé menaçant (le premier cas était une suspicion d´inceste de la part du père, et le deuxième était une maltraitance physique et morale intrafamiliale suite à l´agression sexuelle subie par l´enfant). Pour les deux autres enfants, ils ont été gardés dans leurs familles avec un suivi sur terrain par le délégué de la protection de l´enfance (DPE). En effet, les procédures de protection ont comme référence principale le code de protection de l´enfance (CPE). Il s´agit particulièrement de l´article 20 concernant les conditions menaçantes pour l´enfant parmi lesquelles figurent l´exploitation sexuelle et l´article 59 qui détaille les mesures de protection disponibles. A noter aussi que dans notre contexte tunisien, le juge est amené aussi dans différentes situations à se baser sur son expérience professionnelle et la jurisprudence. Il serait de même important à signaler que le CPE parle d´ « enfant en danger » et non d´ « enfant victime » ce qui qualifie les décisions prononcées par le juge de la famille comme étant des mesures de protection et non réparation de dégât quel que soit sa nature. Concernant les procédures pénales à l´encontre de l´agresseur, l´affaire était suivie par le juge de l´enfance si l´agresseur est mineur. Par ailleurs, si l´agresseur est adulte, ce qui est le cas de la majorité de nos dossiers, l´affaire était suivie par le tribunal et le juge de majeur. En outre, aucune mesure pénale n´a été prise. Le crime n´a pas été confirmé car dans la plupart des cas, les versions rapportées par l´enfant étaient multiples et la désignation des agresseurs était difficile. Il nous a été difficile de poursuivre notre étude auprès du juge de majeurs car l´archive des dossiers se fait selon un autre codage différent de celui utilisé auprès du bureau du juge de la famille.

## Discussion

Concernant les caractéristiques sociodémographiques, la moyenne d´âge des victimes dans la présente étude était de 10 ans ± 3,9. De nombreuses études rapportaient des moyennes d´âge variant entre 10 et 12 ans l'âge auquel la majorité des victimes subissent l´agression sexuelle, avec un pic à 6 ans et un deuxième pic à 13-14 ans [3,4]. De même, la prédominance féminine observée dans notre étude était constatée dans d´autres travaux. Une étude rétrospective réalisée à partir des registres juridiques des affaires d´agressions sexuelles commises dans une région du Centre-Est tunisien jugées de 1993 à 1998 a révélé que 629 affaires ont été recensées, correspondant à une incidence de 14,7 victimes pour 100 000 habitants par an, 81 % des victimes étaient mineures et 58 % de sexe féminin [3]. Nous avons relevé que la totalité des mineurs qui ont été colligés vivaient dans un milieu urbain. Ce chiffre peut être expliqué par la difficulté d´accessibilité des régions rurales à la justice et les longues procédures contraignantes que ces habitants peuvent rencontrer mais aussi que ce sujet reste encore tabou dans ces régions. En outre, un milieu familial défavorable sur les plans socio-économique, relationnel et affectif représente un facteur de vulnérabilité selon la majorité des études [5].

Pour la nature des sévices sexuels, notre étude a révélé que les attouchements sexuels et les pénétrations anales et /ou vaginales étaient les plus fréquents. Ces résultats étaient concordants avec les données de la littérature. D´après une étude plus récente de Silva. W. D en 2016, la pénétration vaginale et anale représentait entre 83% et 87% des cas de sévices sexuelles [6]. Pour les données nationales en Tunisie, selon le rapport de délégué général de la protection de l´enfance montrent [7]: des agressions sexuelles signalées étaient dans 50.5% des attouchements sexuels, 35.5% des rapports sexuels. S´agissant des caractéristiques de l´agresseur, ce dernier était dans la majorité du cas un homme adulte (43%) de l´entourage de la victime (54%) et 3/4 des abus sont commis par l´entourage familial. Ce résultat rejoint les données des différentes études, avec une proportion d'abuseurs de sexe masculin variant, selon les études entre 90 et 98% [4,8]. De plus, la majorité des agresseurs sexuels des enfants sont des adultes entre 35 et 40 ans ou des adolescents [9,10] et selon Wilken, dans son enquête menée sur des adultes victimes d´abus sexuels à l´enfance, l´agresseur était un membre de la famille dans 54% de cas et une personne de l´entourage dans 26% de cas [11]. Concernant la fréquence de l´agression, elle était unique dans 47% des cas et répétée dans 44% des cas. En accord avec nos résultats, des auteurs ont prouvé que 8 fois sur 10 les abus sont répétés [4]. Suite à l´agression sexuelle, L´association à d´autres facteurs de stress, rapportée par certaines victimes de l´étude est classique selon les données de la littérature. Pour notre étude, une symptomatologie initiale dominée par des manifestations anxio-dépressives (trouble dépressif 32%, trouble de l´adaptation avec anxiété 21%, trouble stress post-traumatique 12%) a été notée. En accord avec notre étude ; une méta-analyse impliquant 25 367 personnes a révélé une association significative entre l’abus sexuel durant l´enfance et la dépression, le suicide, l'état de stress post-traumatique (ESPT) et le vagabondage sexuel [12].

Le suivi à court et moyen terme a été marqué essentiellement par les perdus de vue. Cela pourrait s´expliquer par le caractère encore tabou du sujet à travers les différents milieux et cultures. Parmi nos victimes, 13% ont souffert d´autres troubles mentaux au cours de suivi. Ils s´agissaient des troubles de conduite graves, violence sexuelle, trouble bipolaire et des éléments psychotiques. Nos résultats étaient en accord avec les données de la littérature. Une large cohorte sur 43 ans menée en Australie a noté que l´exposition à un abus sexuel à l´enfance augmentait significativement le risque de développer des troubles psychiatriques par rapport aux témoins à type de troubles de l´humeur, trouble anxieux, toxicomanie, trouble psychotique et trouble de la personnalité [13]. Concernant les suites juridiques et l´aspect légal, parmi nos victimes, 26,8% ont contacté le bureau de la délégation de protection de l´enfance et seulement 7,5% des enfants ont bénéficié d´un suivi judiciaire avec le juge de la famille. Ce chiffre pourrait être expliqué par la réticence importante de la famille par rapport à la révélation de ce secret et au suivi juridique surtout dans le cas d´un inceste [14]. Cette réticence a des origines solides de type religieux et culturel dans la société. Par ailleurs, face à cette problématique, la question de crédibilité est un vrai défi pour le médecin psychiatre et pour le juge au terme des propos du mineur ou autre intervenant. La suggestibilité peut être double chez le mineur, induite par le parent, par l´enquêteur et par l´examinateur. Elle rend cette mission encore plus difficile. Ceci a été démontré par les expériences de Ceci *et al*. en 1987 [15]. Le degré de sincérité et de véracité des propos de l´enfant nécessite une vigilance importante ainsi qu´une enquête minutieuse qui pourrait prendre assez du temps, chose qui a été objectivée dans notre étude. Du côté du clinicien, cela nécessite une expérience et délicatesse dans la pratique pédopsychiatrique afin de pouvoir analyser parallèlement plusieurs dimensions développementale, socioculturelle et légale.

## Conclusion

L´abus sexuel est un champ très vaste des agressions de nature et d´intensité très diverses. Même Si l´impact physique de cet acte peut manquer dans certaines formes d´abus sexuel, l´impact émotionnel et psychologique est omniprésent avec des expressions cliniques variables. Il s´agit d´une hypothèse à évoquer devant tout changement du comportement, des jeux sexués, troubles sphinctériens… d´apparition brutale et de façon inexpliquée. Désormais nous faisons face à de nouvelles formes d´exploitation sexuelle (cyber agression, “Jihed sexuel”…). Les enjeux culturels, sociodémographiques et économiques doivent être pris en considération dans l´analyse de tout travail traitant ce sujet dans ses différentes dimensions.

### Etat des connaissances sur le sujet

- Les abus sexuels constituent actuellement un véritable drame socioculturel;
- Ce crime est très souvent vécu par l´enfant dans le silence et le secret; l´abuseur est le plus souvent une personne « connue » et familière à l´enfant;
- La symptomatologie relevée chez ces victimes est souvent riche et exprimée de différentes manières.

### Contribution de notre étude à la connaissance

- Nous avons pu constater à travers notre enquête, qu´en Tunisie, du dévoilement au jugement, le parcours de l´enfant victime d´abus sexuel est long et éprouvant. La parole dite en confidence devient objet d´interrogatoires successifs, d´expertises médicales et psychiatriques, justifiés par la recherche des preuves. Ceci peut renforcer l´impact traumatique et décourager le suivi;
- L´amélioration de la prise en charge des victimes d´abus sexuels passe nécessairement par la mise en place de structures spécialisées dans la prise en charge des abus sexuels où les victimes pourront bénéficier d´une assistance médicale, psychologique et juridique gratuite;
- Il faut développer la prévention primaire à partir d´une sensibilisation plus large de la population générale et la mise en place d´un encadrement juridique plus répressif.
